# Hypersensitive Ethylene Signaling and *ZMdPG1* Expression Lead to Fruit Softening and Dehiscence

**DOI:** 10.1371/journal.pone.0058745

**Published:** 2013-03-20

**Authors:** Min Li, Yanmin Zhang, Zongying Zhang, Xiaohao Ji, Rui Zhang, Daliang Liu, Liping Gao, Jing Zhang, Biao Wang, Yusen Wu, Shujing Wu, Xiaoliu Chen, Shouqian Feng, Xuesen Chen

**Affiliations:** 1 State Key Laboratory of Crop Biology, Shandong Agricultural University, Tai'an, Shandong, China; 2 College of Life Sciences, Shandong Agricultural University, Tai'an, Shandong, China; 3 State Key Laboratory for Biology of Plant Diseases and Insect Pests, Institute of Plant Protection, Chinese Academy of Agricultural Sciences, Beijing, China; Wuhan University, China

## Abstract

‘Taishanzaoxia’ fruit rapid softening and dehiscence during ripening stage and this process is very sensitive to endogenous ethylene. In this study, we cloned five ethylene signal transcription factors (*ZMdEIL1, ZMdEIL2, ZMdEIL3, ZMdERF1* and *ZMdERF2*) and one functional gene, *ZMdPG1*, encoding polygalacturonase that could loose the cell connection which associated with fruit firmness decrease and fruit dehiscence to illustrate the reasons for this specific fruit phenotypic and physiological changes. Expression analysis showed that *ZMdERF1* and *ZMdEIL2* transcription were more abundant in ‘Taishanzaoxia’ softening fruit and dehiscent fruit and their expression was inhibited by an ethylene inhibitor 1-methylcyclopropene. Therefore, *ZMdERF1* and *ZMdEIL2* expression were responses to endogenous ethylene and associated with fruit softening and dehiscence. *ZMdPG1* expression was induced when fruit softening and dehiscence but this induction can be blocked by 1-MCP, indicating that *ZMdPG1* was essential for fruit softening and dehiscence and its expression was mediated by the endogenously occurred ethylene. *ZMdPG1* overexpression in *Arabidopsis* led to silique early dehiscence while suppressing *ZMdPG1* expression by antisense *ZMdPG1* prevented silique naturally opening. The result also suggested that *ZMdPG1* related with the connection between cells that contributed to fruit softening and dehiscence. *ZMdERF1* was more closely related with ethylene signaling but it was not directly regulated the *ZMdPG1*, which might be regulated by the synergic pattern of ethylene transcription factors because of both the ZMdERF1 and ZMdERF2 could interact with ZMdEIL2.

## Introduction

Fruit softening and dehiscence greatly reduce commercial value by influencing the fruit taste, flavor, out-looking and shelf life, which is common for certain apple cultivars, including Red Delicious Golden Delicious and ‘Taishanzaoxia’ [1]–[3]. It is especially the case for the apple cultivar ‘Taishanzaoxia’, which suffers from this fruit quality deterioration severer than any other apple cultivars, and which is softening very fast, accompanying with the fruit dehiscence during the fruit ripening [2]. The defect of this cultivar make it an ideal material for dissecting the mechanism underlying the easy softening and dehiscence, which is important for uncovering the mechanism for fruit quality formation, postharvest physiology and fruit breeding.

Previous research indicated that fruit softening was tightly connected with ethylene biosynthesis. Acceleration of fruit softening connect with the rapidly increase of ethylene production in some apple cultivars, so ethylene played an important role in this process [4], [5]. The findings in our group as well as many other international colleagues showed that 1-MCP treatment, which blocked ethylene biosynthesis, effectively prevented fruit softening and dehiscence, strongly demonstrating that ethylene was involved in this process [6]–[9]. In addition, the endo-ethylene accelerate the dehiscence process of flower organ even through ethylene doesn't initiate dehiscence in *Arabidopsis* [10].

Ethylene biological effects are discovered through the ethylene signaling pathway. Firstly, ethylene is perceived by the target cells through receptors (*ETRs*). Subsequently, the signal transmission would be regulated by the ethylene signaling negative regulator *CTR1* (constitutive triple response 1) and positive regulators *EIN2* (ethylene insensitive 2) and *EIN3* (ethylene insensitive 3). In the end, the signal would be transmitted to ethylene responsive transcription factors(*ERFs*) [11].

*EIN3*/*EILs*(*EIN3-like* genes) belongs to a small transcription factors family including several DNA-binding domains such as acidic domain, proline-rich and basic domains[12], [13]. *EIN3* gene is firstly identified from *ein3* mutants of *Arabidopsis*. Subsequently, four *LeEILs* response for fruit ripening have been isolated from tomato [13], [14]. Antisense suppression of *LeEILs* reveals functional redundancy in tomato [13]. The function of *EIN3/EILs* is demonstrated at the protein level, the DNA-binding protein of this family directly binds to the primary response element in promoter of *ERF1* (Ethylene response factor) to regulate *ERF1* expression in *Arabidopsis* [15]. *EIL* genes have been also isolated from fruits such as tomato, melon, kiwifruit and apple [16]–[20]. In transgenic apple, *MdEILs* activate the *MdPG1* promoter in the presence of ethylene [19]. *CmEIL1* and *CmEIL2* as ripening-related genes regulate the transcription of *CmACO1*in ripening melon fruits [16]. It is similar that *AdEIL2* and *AdEIL3* activate the expression of ripening-related genes *AdACO1* and *AdXET5* in kiwifruit [21]. However, much less is known response for *EILs* function in transcriptional level in fruit.

*ERFs* belongs to the large AP2/ERF superfamily including 122 members in *Arabidopsis* and 139 members in rice [22], and contains two conversed DNA-binding domains YRG element and RAYD element [23]. Referred to as the ethylene responsive element binding proteins (EREBPs), *ERF* was first isolated from tobacco by binding to the GCC motif in the promoter of functional genes [24]. Then, four *ERF* genes were identified which induced fruit ripening in tomato. Transgenic experiment result showed that antisense *LeERF1* under the control of *CaMV35* with longer postharvest life [25], and *SlERF*2 was shown to express predominantly in ripening fruits [26]. All four *LeERFs* have the ability binding to GCC-box element present in several defense responsive genes [27]. In kiwifruit, AdERFs protein did not bind to the *AdEXP1* promoter containing a GCC-box, but the activation of *AdXET5* was significantly suppressed by AdERF9 in interaction experiments which suggesting that fruit ripening might be regulated by unknown mechanism. Two *MdERF* genes had been isolated from ripening fruit which were regulated by ethylene [28]. Same as *SlERF2*, *MdERF2* expressed exclusively in ripening fruit, and *MdERF1* was expressed predominantly in ripening fruit. However, there is little research involved in the function of *MdERF1* and *MdERF2* in fruit, and the role of *MdEILs* and *MdERFs* is unknown.

Besides the ethylene signaling, another set of important ingredients correlate with softening and dehiscence are hydrolytic enzymes located at the cell wall, including PGs, because in essence the loosened or even broken cell connection cause fruit softening and dehiscence [29], [30]. *PG* was first cloned from ripening tomato cDNA library [31]. In tomato fruit, a correlation between endo-PG activity and softening has been observed in a number of cultivars [32], [33]. However, endo-PG activity in transgenic tomato plants is not the sole determinant of fruit softening [34], [35]. The relationship between *PG* and fruit softening has been presented in other plant species, such as apple, pears, kiwifruit and peach and certain *PGs* are also accountable for the organ abscission or dehiscence [36]–[39]. For example, repression of *PG1* in apple brings about firmer fruit [40]. In ‘Gold Delicious’, softening is closely depended on the expression of *MdPG* with comparison of ‘Fuji’ [5]. *PG* overexpression in transgenic apple lead to premature leaf shedding because cell adhesion is reduced in leaf abscission zones [41]. *MdPG1* is repressed in transgenic ‘Royal Gala’ apples while returned to wild-type levels with ethylene treatment [19]. Further, a MdEIN3-like transcription factor activates the promoter of *MdPG1* by transient assays [19]. In addition, dehiscence occurs in wild siliques but not in *adpg1 adpg2 qrt2* triple mutants in *Arabidopsis* [42].

In this research we characterized the *ZMdERFs*, *ZMdEILs* and *ZMdPG1* in ‘Taishanzaoxia’ with the aim to uncover the heavily occurred softening and dehiscence. In this study, the expression of ethylene transcription factors and *ZMdPG1* was significantly high and showed ripen-inducible pattern while their expression in ‘Liaofu’ was relatively stable and did not obvious change during fruit growth and developmental period. The *ZMdPG1* induction was mediated by endogenously biosynthesized ethylene. BiFC assay showed that ZMdEIL2 interact with ZMdERF1 and ZMdERF2, respectively. Transgenic analysis showed that *ZMdPG1* overexpression could result in cell connection broken as demonstrated by the silique dehiscence of *ZMdPG1* overexpressed *Arabidopsis* transgenic plant. Therefore, *ZMdPG1* expression may lead to fruit dehiscence in ‘Taishanzaoxia’, which is induced by ethylene.

## Results

### Ethylene promote the loss of fruit firmness

**Figure 1:**
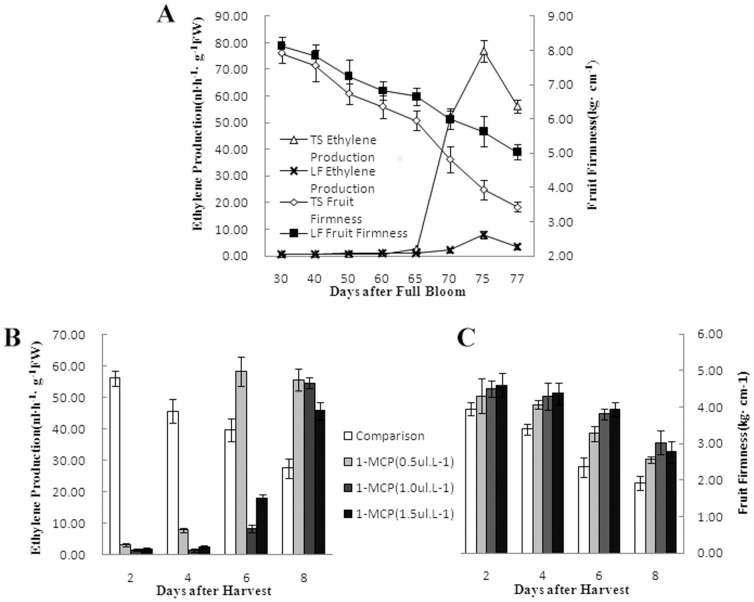
The comparison of ethylene production and fruit firmness in ‘Taishanzaoxia’ and ‘Liaofu’ cultivar. (A) Changes of fruit firmness and ethylene production in different apple cultivars during fruit development. (B) Effect of different levers 1-MCP on ethylene production in ‘Taishanzaoxia’. (C) Effect of different levers 1-MCP on fruit firmness in ‘Taishanzaoxia’. TS represent ‘Taishanzaoxia’ cultivar. LF represents ‘Liaofu’ cultivar.

Fruit softening was closely associated with ethylene production [43]. As shown in Figure 1, there was a sharp increase of ethylene production in ‘Taishanzaoxia’, and the decline of fruit firmness was accelerated by the rapid collection of ethylene production that led to significant diversity in fruit firmness between ‘Taishanzaoxia’ and ‘Liaofu’. The change of fruit firmness was similar from 30 d to 60 d in two cultivars, whereas they assumed the difference after 65 d when ethylene biosynthesis began to increase in ‘Taishanzaoxia’ (Figure 1). The rapid increase of ethylene production might result in the fruit softening. After harvest, ‘Taishanzaoxia’ fruits were treated with 1-MCP. Fruit softening behavior was clearly limited associate with the significant inhibition of ethylene production. Compared with the control, fruit firmness of treatment was inhibited clearly at 6 d after harvest (Figure 1). Fruit dehiscence was also observed with the abundance of ethylene over the postharvest period.

### Cloning and analysis of *ZMdERFs*, *ZMdEILs* and *ZMdPG1*

cDNA synthesized from ‘Taishanzaoxia’ ripening apple fruit was used as a template for RT-PCR, and the primers were designed from the nucleotide sequence among ‘Gold Delicious’ *MdERFs*. Then two cDNA full-length fragments corresponding to *MdERF1* and *MdERF2* were cloned and named as *ZMdERF1* and *ZMdERF2* (*ZMdERF1*, GenBank accession number KC128856; *ZMdERF2*, GenBank accession number KC128857). Alignments of amino acid showed both two genes contained the YRG and RAYD elements [23] (Figure S1A) indicating that they were the ethylene transcription factors in ‘Taishanzaoxia’. Phylogenetic analysis revealed that ZMdERF1 and ZMdERF2 were in the same cluster with ‘Gold Delicious’ MdERF1 and MdERF2, respectively (Figure S1B).

Three EIN3-like genes named *MdEIL1*, *MdEIL2* and *MdEIL3* (*MdEIL1*, GenBank accession number KC128859; *MdEIL2*, GenBank accession number KC128859; *MdEIL3*, GenBank accession number KC128860) were amplified from ‘Taishanzaoxia’ using the same strategy. They had the high similarity with each other (Figure S2A), sharing the commonly conserved domain with that of tobacco, Kiwifruit and tomato, which included a high acidic region, five basic domains and a proline-rich domain [12] (Figure S2B).

Functional gene *ZMdPG1* (*ZMdPG1*, GenBank accession number KC128861) gene was also cloned using ‘Taishanzaoxia’ ripening fruit cDNA. *ZMdPG1* gene encoded 460 amino acids. The predicted ZMdPG1 protein shared high similarity with known PGs in ‘Gold Delicious’ apple, pear, peach, kiwifruit and *Arabidopsis* (Figure S3A). NCBI (National Center of Biotechnology Information) assay indicated the homology between *ZMdPG1* and *ADPG1* was 67%. Phylogenetic analysis revealed that ZMdPG1 was in the same cluster with pGDPG-1 from ‘Gold Delicious’ cultivar and there was only two amino acid difference between them. Z*MdPG1* was close to *Arabidopsis ADPG1* and *ADPG2* (Figure S3B).

*ZMdQP*, the promoter region of *ZMdPG1*, (*ZMdQP*, GenBank accession number KC128862) was identified in ‘Taishanzaoxia’ using high-TAIL PCR. Sequence analysis revealed that the *ZMdQP* was approximately 1.7 kb in length from the *ZMdPG1* start cordon. The sequence 787-1697 bp in *ZMdQP* was high similar with the promoter of *pGDGP-1* while there was significant difference in 1–786 bp (Figure S4). 29 elements in *ZMdQP* were analyzed, such as A-box, AT-rich element, CCGTCC-box and CGTCA-motif were involved in regulatory function, elicitor-mediated activation, meristem specific activation and MeJA-responsiveness. The traits TATA-box and CAAT-box at -303 bp and -244 bp from the ATG start cordon were identified in the upstream region.

We put the sequence of the genes cloned in our research into GDR (Genome Database for Rosaceae) database to compare with the apple genome. Many homologues were found in the apple genome (Table S1).

### Expression profile of *ZMdERFs*, *ZMdEILs* and *ZMdPG1*

**Figure 2:**
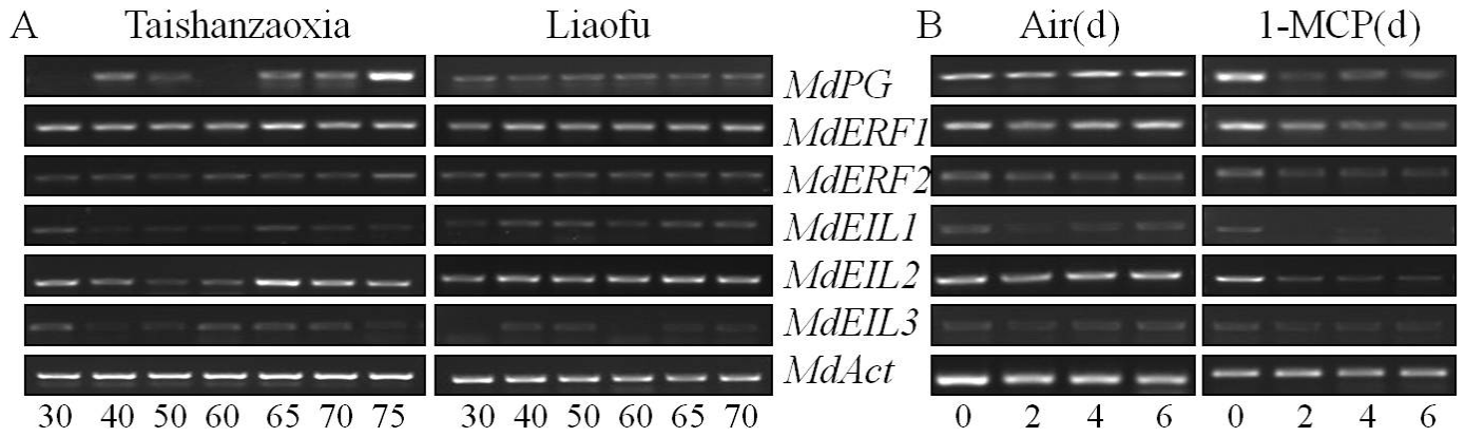
The expression pattern of *ZMdPG1* and ethylene signal transcription factors. (A) Expression of the *ZMdPG1* and ethylene signal transcription factors during fruit development. Data obtained from ‘Taishanzaoxia’ and ‘Liaofu’ apple fruit are shown in order from left to right. Data for different ripening stages are shown in order from left to right. Numbers below each lane indicate the number of ripening days after full bloom. Data from three repeats are provided. (B) Expression of the ZMdPG1 and ethylene signal transcription factors in ‘Taishanzaoxia’ apple fruit after harvest. Fruit were held at 24°C and treated with air (Air) or 1.0 µl.L-1 1-MCP (1-MCP) for 24 h. Data are shown in order from left to right. Data for different stages are shown in order from left to right. Numbers below each lane indicate the number of days after harvest. Data from three repeats are provided.

To understand the molecular mechanism of fruit softening and dehiscence, the expression of *ZMdPG1* gene and five ethylene signaling components *ZMdERF1*, *ZMdERF2*, *ZMdEIL1*, *ZMdEIL2* and *ZMdEIL3* were investigated. As shown in Figure 2, Z*MdERF1* was constitutively expressed during the development of ‘Taishanzaoxia’ and ‘Liaofu’ fruits but its abundance in ‘Taishanzaoxia’ was higher than that in ‘Liaofu’. Z*MdEIL2* also showed a ripen-inducible trend in soften ‘Taishanzaoxia’ fruits while this trend was not obvious in ‘Liaofu’. In addition, the transcription of Z*MdERF1* and Z*MdEIL2* was more abundant in ‘Taishanzaoxia’ associated with the abundance of ethylene production in 65 d, whereas their expression in ‘Liaofu’ did not have obvious change during fruit development (Figure 2A). The expression of Z*MdERF2* increased a little in the ripening and softening fruit of ‘Taishanzaoxia’. With 1-MCP treatment, fruit softening and dehiscence, ethylene production and the expression of ethylene-induced genes were suppressed. Transcript levels of Z*MdPG1*, Z*MdERF1*, Z*MdEIL1* and Z*MdEIL2* were substantially inhibited compared with control which indicated that the transcription of four genes was closely connected in ethylene during fruit softening and dehiscence stages. 1-MCP treatment has little effect on the reduction of Z*MdERF2* and Z*MdEIL3* (Figure 2B). Unlike in ‘Taishanzaoxia’ fruits, Z*MdEIL2* and Z*MdPG1* expressions in ‘Liaofu’ fruit maintained a same level during the whole growth and development period.

**Figure 3:**
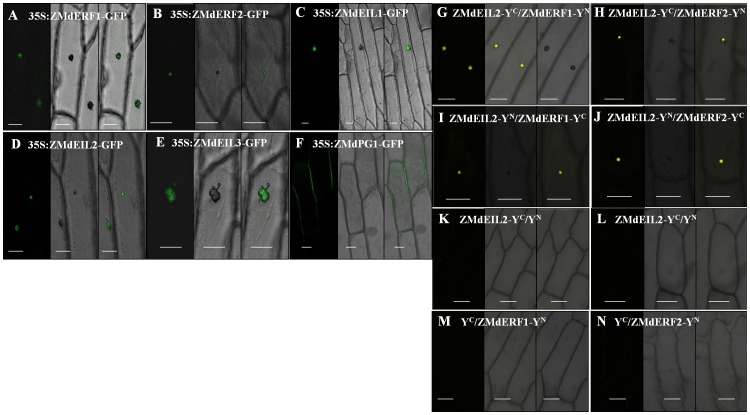
Functional characterization of ZMdEILs, ZMdERFs and ZMdPG1. (A) ZMdEIL2 interacts with ZMdERF1and ZMdERF2 *in vivo* in the BiFC assay. YFP fluorescence signals are detected in 2 d. (B) Transient expression assays showed that ZMdPG1 protein localized in the cell wall. ZMdERF1, ZMdERF2, ZMdEIL1, ZMdEIL2 and ZMdEIL3 localize in the cell nucleus. GFP fluorescence signals are detected in 2 d. bar  = 50 µm

To confirm the functions of *ZMdERF1*, *ZMdERF2*, *ZMdEIL1*, *ZMdEIL2* and *ZMdEIL3* in cell, those five genes fused with the *GFP* gene under control of the *CaMV35S* promoter were transferred into onion epidermal cell. Localization of the fusion protein was determined by visualization with a confocal microscope. We found that ZMdERF1, ZMdERF2, ZMdEIL1, ZMdEIL2 and ZMdEIL3 proteins were accumulated in cell nucleus (Figure 3A)

### Overexpression of *ZMdPG1* in transgenic plants

**Figure 4:**
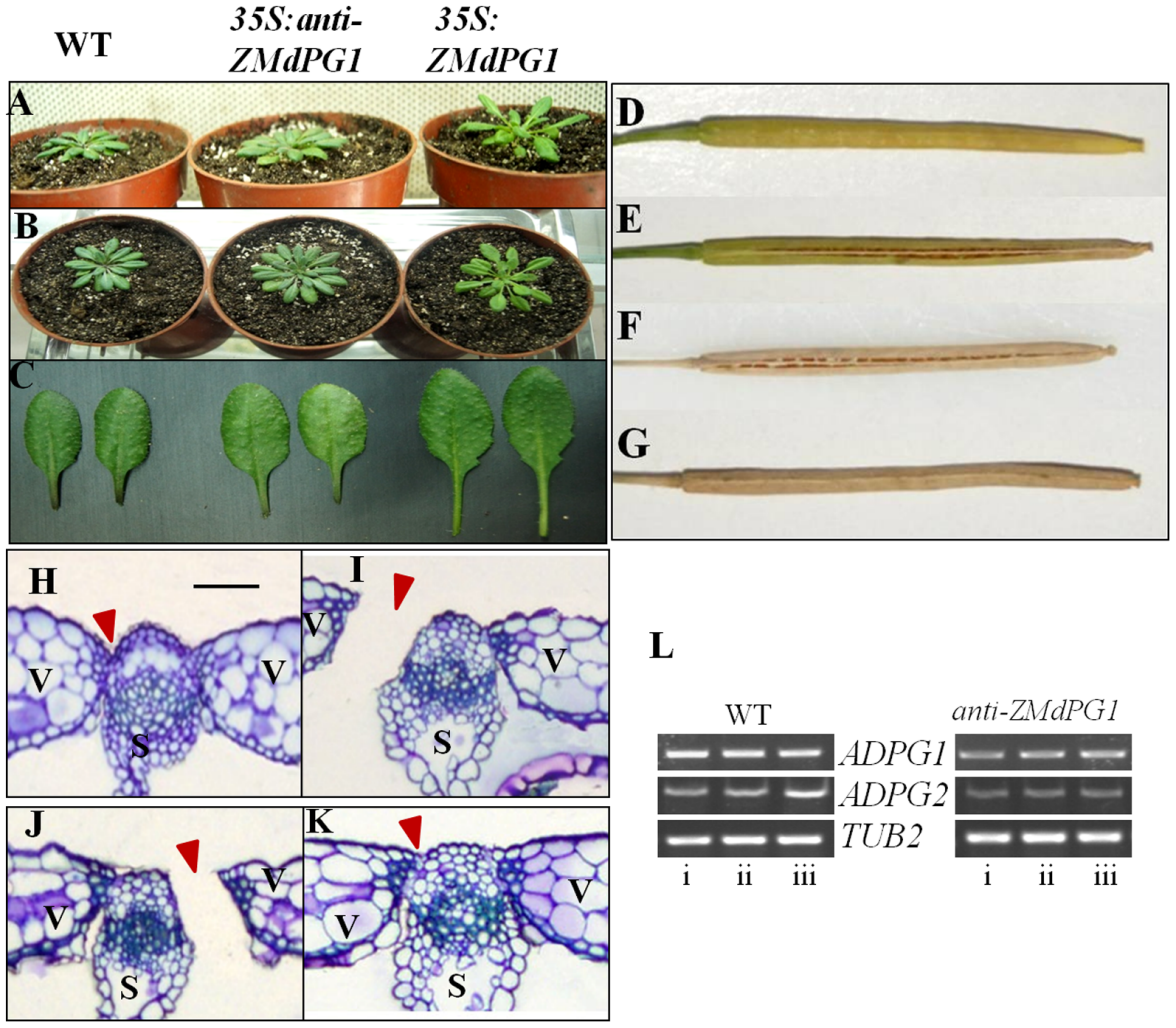
The functional pattern of ZMdPG1 in transgenic Arabidopsis. The plants showing loosened, slant growth and long petiole phenotype in overexpressed ZMdPG1 Arabidopsis but not in antisense Arabidopsis. Silique dehiscence and cell separation occurred in faint yellow silique of overexpressed ZMdPG1 Arabidopsis while that occurred in mature and dry wide siliques but not in antisense transgenic Arabidopsis. Triangle represents DZ. (A) The profile of phenotype. (B) The profile of phenotype. (C) The phenotype of petiole (D) The faint yellow silique of wild Arabidopsis, there is not split (early of stage 18); (E) The faint yellow dehiscence silique of transgenic Arabidopsis containing 35 S:ZMdPG1 (early of stage 18); (F) The mature and dry dehiscence silique of wild Arabidopsis (stage 19); (G) The mature and dry silique of transgenic Arabidopsis containing anti-ZMdPG1 (stage 19); (H) Transverse section of wide-type stained with Toluidine blue corresponding to (D); (I) Transverse section of overexpressed transgenic Arabidopsis stained with Toluidine blue corresponding to (E); (J) Transverse section of wide-type stained with Toluidine blue corresponding to (F); (K) Transverse section of antisense transgenic Arabidopsis stained with Toluidine blue corresponding to (G). Arrowheads indicate the DZ, bar = 50 µm; (L) The expression pattern of ADPG1 and ADPG2 in antisense ZMdPG1 transgenic Arabidopsis. (i) developing siliques in stage 17, (ii) yellow siliques in stage 18, (iii) fully matured siliques in stage 19.

To understand the biological role of *ZMdPG1*, the sense and antisense *ZMdPG1* transgenic *Arabidopsis* under the control of *CaMV35* promoter were performed. As shown in Figure 4. Most overexpressed *ZMdPG1* plants displayed similar phenotype such as loosened and slant growing habit, strait-angled branches and longer petioles. In contrast, Antisense *ZMdPG1* plants showed the tight and regular phenotype, leaves grew parallel to and horizontal plane and petioles was short. In addition, the overexpression of *ZMdPG1* in *Arabidopsis* resulted in earlier dehiscence fruit. Overexpressed *ZMdPG1 Arabidopsis* revealed split siliques in early stage 18 (Figure 4E), while that was observed in stage 19 in wide-type control plants (Figure 4F). It was in coincidence with the report that silique dehiscent normally in stage 19 in wide *Arabidopsis* [44]. But there was not dehiscence in antisense transgenic siliques (Figure 4G). Semi-quantitative RT-PCR analysis showed that the expression of *ADPG1* and *ADPG2* in antisense *ZMdPG1* transgenic *Arabidopsis* were less than in wide type. The *ADPG1* expression was partly inhibited and the *ADPG2* expression was significantly inhibited (Figure 4L).The result was further demonstrated by organization experiment. Cross sections revealed that cell separation occurred in the DZ (Dehiscence Zone) in overexpressed *ZMdPG1* siliques when siliques turned light yellow (Figure 4I). The same phenomenon occurred in mature and dry wide siliques (Figure 3J) but not in antisense transgenic plants (Figure 4K). The result indicated that dehiscence was caused by cell separation in the DZ. In the protein location assay, we found that the expressed ZMdPG1-GFP fusion protein was precisely localized at the cell wall (Figure 3A) which suggested that the expressed *ZMdPG1* sited at the cell wall could play an important role in promoting fruit dehiscence.

**Figure 5:**
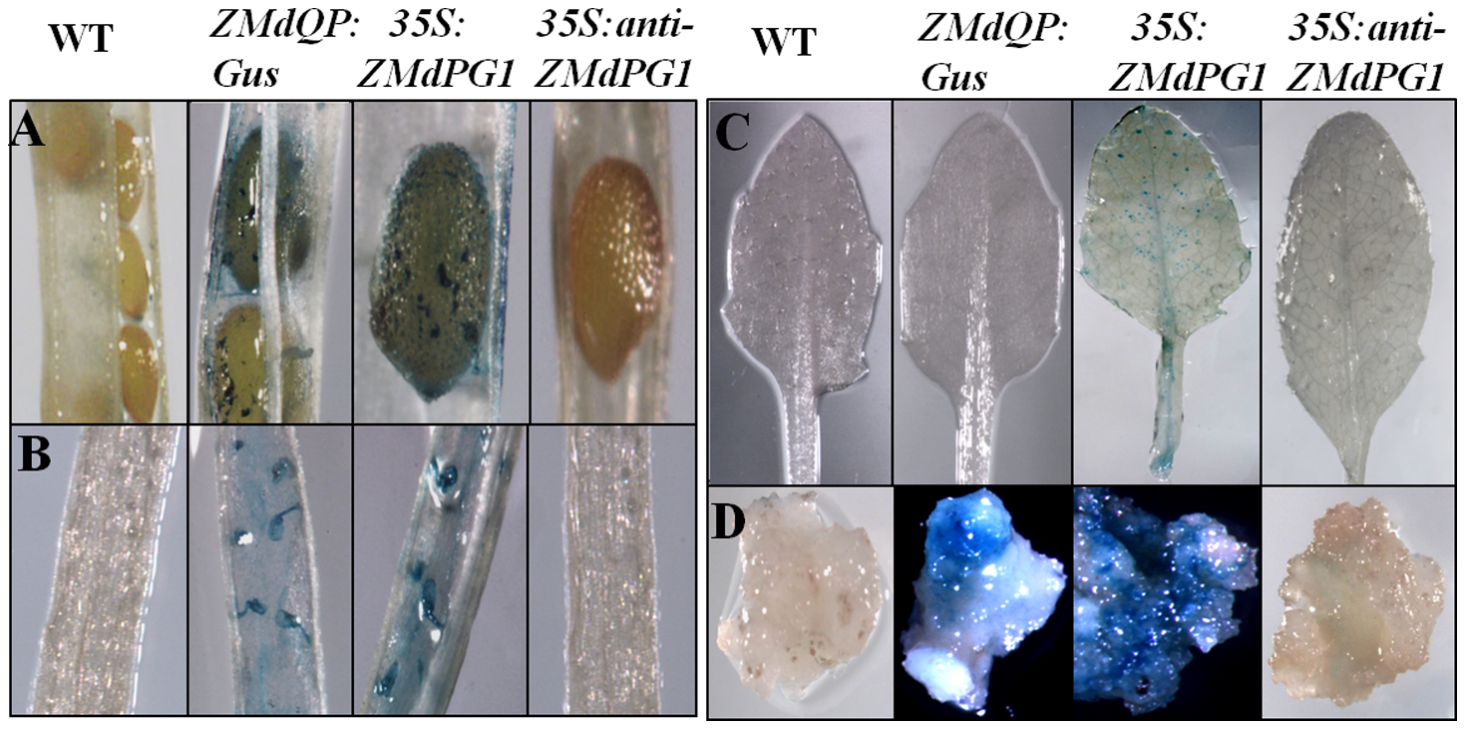
The expression pattern of ZMdPG1 in transgenic Arabidopsis and cell callus. The expression of ZMdPG1 is detected in silique valve DZs (A), seeds (A), ovule funiculus (B) and apple calli (D). The overexpression of ZMdPG1 is detected in leaf and petiole of Arabidopsis (C) and apple callus (D).

To investigate *ZMdPG1*expression pattern, the sense and antisense tissues showing GUS staining under the control of *CaMV35* promoter were developed in transgenic *Arabidopsis* and transgenic apple callus, and the construction of *ZMdQP* fused with the *GUS* gene was also performed in transgenic *Arabidopsis* and transgenic apple callus. As shown in Figure 5. GUS signaling was detected in matured silique valve DZs, seeds and ovule funiculus in overexpressed *ZMdPG1* plants and ZMdQP-GUS plants (Figure 5A–B). GUS signaling was also observed in leaf and petiole in overexpressed *ZMdPG1* plants (Figure 5C). Further more, overexpressed GUS signals were detected in few region of apple callus while ZMdQP-GUS signals were observed only in part regions of apple callus (Figure 5D).

### ZMdEIL2 interacts with ZMdERF1 and ZMdERF2 physically

In this study, BiFC [45] assays were performed to investigate the function of *ZMdEILs* and *ZMdERFs*. *ZMdEIL1*, *ZMdEIL2*, *ZMdEIL3*, *ZMdERF1* and *ZMdERF2* were fused with the N-terminal fragment and C-terminal fragment of yellow fluorescent protein (YFP), respectively. As a result, strong yellow fluorescent signals were observed in cells containing ZMdERF1/ZMdEIL2, ZMdEFR2/ZMdEIL2 (Figure 3B). Meanwhile, there were not fluorescent signal was observed in other combinations and in control. The results indicated that ZMdERF1 and ZMdERF2 interacted with ZMdEIL2 specifically in cells.

### ZMdERF1 did not bind to the *ZMdPG1* promoter

**Figure 6:**
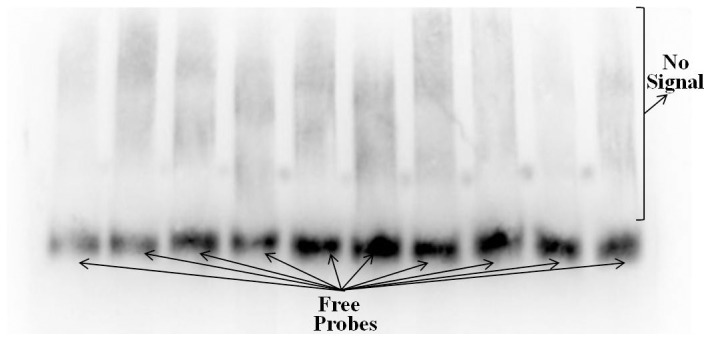
ZMdERF1 doesn't directly regulate the promoter of the ZMdPG1. Ten probes were performed. Bracket indicates where binding signal has not occurred.

To examine whether the nuclear protein ERF1 cloud bind to *ZMdQP*, electrophoretic mobility shift assay (EMSA) was performed. The recombinant His-ZMdERF1 protein was induced by isopropyl-β- D-thiogalactopyranoside (IPTG) and identified *via* Sodium dodecyl sulfate-polyacrylamide gel electrophoresis (SDS-PAGE). Then abundant His-ZMdERF1 protein purified with His Trap TM FF crude [46]. In addition, ten overlapping fragments covering 1.7 kb upstream sequence from the *ZMdPG1* translation initiation site were labeled with biotin for chemiluminescence. But slower migrating band was not observed after ten probes were incubated with ZMdERF1 protein (Figure 6). The result demonstrated that ZMdERF1 protein did not bind to *ZMdQP.*

## Discussion

Fruit softening and dehiscence are greatly blocked fruit quality formation and fruit breeding. In this research, the ‘Taishanzaoxia’ cultivar provides a good model for uncovering the molecular mechanism because of the easy softening and dehiscence. Previous work suggested that ‘Taishanzaoxia’ fruit was sensitive to endogenous ethylene [2], [8]. Unlike many other firmness apple fruits such as ‘Liaofu’ and ‘Fuji’ which had little ethylene production [28], the fruit initially and rapidly soften of ‘Taishanzaoxia’ species followed by a ethylene burst. After treatment of 1-MCP, ‘Taishanzaoxia’ fruit ethylene production was significantly inhibited which associated with clear limit of fruit softening behavior (Figure 1).Those results suggested that the loss of ‘Taishanzaoxia’ apple fruit firmness had a strong response to ethylene biosynthesis and it was sensitive to endogenous ethylene. Compared with control, the fruit dehiscence was inhibited with little ethylene biosynthesis after the 1-MCP treatment which also suggested that it was sensitive to endogenous ethylene. So we supported the hypothesis that the regulation for ethylene to the physiological change in fruit firmness and dehiscence.

To investigate the role of *ZMdERFs* and *ZMdEILs*, the feature of five ethylene signal factors was confirmed firstly. In this research, *ZMdERF1* and *ZMdERF2* showed high similarity at conserved region and shared two characteristic ERF elements, YRG and RAYD (Figure S1). Three *EIL* genes, named *ZMdEIL1*, *ZMdEIL2* and *ZMdEIL3* which showed high identity with the *EILs* in other plant species (Figure S2). *ZMdPG1* has the high similarity with the known *PG1* (Figure S3). WAAEIRD box, α-helix and β-sheet [47] were also observed in YRG and RAYD elements of *ZMdERFs*. However, little was known about the function of YRG and RAYD element in apple fruit. In *Arabidopsis*, YRG bound to DNA *via* its β-sheet for the highly basic in this region [47]. The α-helical structure in RAYD might interact with the major groove of DNA or regulated protein–protein interactions [23]. It was inferred that ZMdERFs might regulated downstream function genes *via* YRG or RAYD elements. It was predicted that the WAAEIRD motifs may be responsible for DNA binding sequence [23]. The conserved structure of *ZMdEILs* from ‘Taishanzaoxia’ fruit including highly acidic region, proline-rich regions but less was known about their function. The similar regions was only described as transcriptional activation domains in *Arabidopsis* [48]. The coil-basic motif was also observed in ZMdEILs which might mediate DNA binding.

*ZMdERF1* gene was one of ethylene transcription factors, whose expression was constitutively higher during ‘Taishanzaoxia’ fruit ripening and softening and it can be induced when ethylene biosynthesis began to increase. This trend fit with those from other species such as ‘Golden Delicious’, where *MdERF1* expression pattern paralleled the ethylene rise in ripening fruit [28]. However, 1-MCP treatment inhibited the increase of *ZMdERF1* expression which was associated with the delay of the loss of fruit firmness and the fruit dehiscence (Figure 2). These results raised the possibility that *ZMdERF1* expression was connect in fruit softening and dehiscence. Another finding was the difference in *ZMdERF2* expression pattern. *ZMdERF2* was very similar in conservative amino acid sequence to *ZMdERF1*, but their expression patterns were different response to ethylene in developing and softening apple. *ZMdERF2* showed little ethylene response, but its expression pattern was stronger than that in ‘Liaofu’ at the late stage of fruit development associated with fruit softening, and with 1-MCP treatment, *ZMdERF2* showed relatively lower expression levels consistency in delayed softening (Figure 2). The result suggested that *ZMdERF1* was likely to be associated with softening in ‘Taishanzaoxia’ fruit. Like the *EIL* genes in ‘Royal Gala’ apple and kiwifruit [19], [21], *ZMdEIL2* gene showed ripen-inducible trend and exhibited response to ethylene, it was constitutively expressed in ‘Taishanzaoxia’ fruit during ripening and harvesting stages. This agreed with the finding of ethylene-dependent activity with *EIL2* in transgenic apple [19]. The result implied that *ZMdEIL2* was necessary for regulation to fruit softening and dehiscence.

Previous reports have confirmed that *ERFs* is considered to have the ability to regulate functional genes, such tomato *LeERFs* [27]. In order to uncover the regulation role of *ZMdERF1*, ripening related gene *ZMdPG1* was selected for promoter isolation. We know that *PG* activity was connected with fruit softening in many species. In this study, we found the high level expression of *ZMdPG1* was not only led to fruit softening but also resulted in fruit dehiscence during ‘Taishanzaoxia’ ripening period. This trend was closely similar with the *ZMdERF1* gene expression. However, the EMSA assay did not proved ZMdERF1 protein has the ability that directly binding to the *ZMdPG1* promoter (Figure 6). It is well established that ERF1 is a GCC-box-binding protein [15], [49]. However, there is not GCC-box element in *ZMdPG1* promoter. The result suggested that *ZMdPG1* was not the target gene of ZMdERF1 protein, and *ZMdPG1* might be activated by some additional regulatory mechanism. In other species, different mechanisms referred to ethylene regulation were demonstrated. In *Arabidopsis*, AtEBP protein interact with a basic *Leu* zipper transcription factor to regulate the expression of functional genes [50]. The activity of *AdXET5* promoter was significantly suppressed by AdERF9 [21]. *MdEIN3-like* transcription factor activated the expression of *MdPG1* in transient assays [19]. *ZMdPG1* from ‘Taishanzaoxia’ fruit might regulate by potential unknown molecular mechanism. BiFC experiment evidenced that ZMdERF1 and ZMdERF2 interacted physically with ZMdEIL2 (Figure 3B), and the transcription of *ZMdERF1* and *ZMdEIL2* was more abundant in ‘Taishanzaoxia’. Such activated assays might suggest that *ZMdPG1* was regulated by the synergic pattern of ethylene transcription factors. The regulation of ethylene signal factors to *ZMdPG1* remains to be determined.

PGs play critical roles in cell separation during plant organ abscission or dehiscence processes, and the regulation of *PG* to fruit softening has been presented in many plant species [29], [34], [42], [43]. In this research, the expression of *ZMdPG1* was observed in softening fruits and showed ripen-inducible pattern. After 1-MCP treatment, the activation of *ZMdPG1* was inhibited and then fruit dehiscence was suppressed. So ‘Taishanzaoxia’ fruit softening and dehiscence were regulated by *ZMdPG1*. For further investigating the role of *ZMdPG1*, the transgenic *Arabidopsis* was provided because of apple transformation is a long process. *ZMdPG1* overexpression in *Arabidopsis* led to seed silique early dehiscence while in antisense *ZMdPG1Arabidopsis*, the expression of *ADPG1* and *ADPG2* which were essential for silique dehiscence were inhibited and silique naturally opening was prevented (Figure 4). The ectopic expression of *ZMdPG1* was involved in cell separation and contributed to fruit dehiscence. Similar results have been shown in other researches. Genetic analysis demonstrates that *ADPG1* and *ADPG2* contribute to silique dehiscence in *Arabidopsis* [42]. *PG* overexpression in transgenic apple resulted in premature leaf shedding [41]. Leaf abscission was delayed when *PGs* were silenced in tomato [51]. These data raised the confirmation that *ZMdPG1* was one of important role which led to apple fruit dehiscence. In addition, *PG* involve in cell wall change resulted in soften fruit and loosened flesh [5], [34]. In this study, the firmness of fruit was obtained when *ZMdPG1* expression was inhibited by 1-MCP treatment. The phenotype of overexpressed *Arabidopsis* was looser than that in control and antisense transgenic plants, and the petiole in sense *Arabidopsis* was longer than that in wide and antisense plants. These results showed that *ZMdPG1* was involved in cell change and the loss of apple fruit firmness. This trend was consistent in pear that the accumulation of *PG* gene was also paralleled with the fruit softening [43]. So *ZMdPG1*expression led apple fruit softening and dehiscence. The primary molecular elucidation for apple fruit dehiscence and softening provided important information for the breeding.

## Conclusion

In this research, we cloned two ethylene signaling components, *ZMdERF1* and *ZMdERF2*, three EIL-likes genes *ZMdEIL1*, *ZMdEIL2* and *ZMdEIL3*, and one *ZMdPG1* gene in the easily soften and dehiscence apple cultivar. *ZMdERF1*, *ZMdEIL2* and *ZMdPG1* expressions were associated with fruit softening and dehiscence. *ZMdERF1* was more closely related with ethylene signaling but it was not directly regulated the *ZMdPG1*, and both the ZMdERF1 and ZMdERF2 could interact with ZMdEIL2. *ZMdPG1* overexpression in *Arabidopsis* led to silique early dehiscence. In contrast, suppressing *ZMdPG1* expression by antisense *ZMdPG1* prevented silique naturally opening. *ZMdPG1* related with the connection between cells that contribute to fruit softening and dehiscence.

## Materials and Methods

### Plant materials

Two apple cultivars ‘Taishanzaoxia’ and ‘Liaofu’ were obtained from fruit Corp. Liaocheng, Shandong, China. To analysis the spatiotemporal expression, apple fruits were picked at different developmental stages. In ‘Taishanzaoxia’, three postharvest treatments with 0.5 µl·L^−1^, 1.0 µl·L^−1^ and 1.5 µl·L^−1^ 1-MCP for 24 h and a control treatment (air) were supplied and stored at 25°C. Ethylene production and fruit firmness were recorded. Four replicates (two fruit for each replicate) were performed to exam ethylene production, and 8 replicates (one fruit for each replicate) were used for fruit firmness.

Callus of ‘Taishanzaoxia’ were induced in vitro on Murashige and Skoog (MS) medium containing 0.6 mg·L^−1^ 6-BA and 0.5 mg·L^−1^ IAA. *Arabidopsis* were cultivated in light incubators under 16/8-h (day/night, 22°C/21°C) photoperiod. 1/2 MS medium was used for selection of transgenic plants.

### Determination of firmness and measurements of ethylene

The firmness of unpeeled apples was measured with the TA.XT plus texture analyzer (Stable Microsystems, Surrey, U.K.) [52]. The ethylene concentration of fruits was tested with a gas chromatograph (Shimadzu, Kyoto, Japan) equipped with a flame ionization detector.

### RNA isolation and gene cloning

RNA was extracted from the fruits and postharvest fruits following the introduction of Bioteke kit (Bioteke, Beijing, China) with DNAse treatment (Fermentas, Hanover, ZMD, USA). Three replicates for each sample were sectioned to reduce the material variability. First-strand cDNA was synthesized using oligo(dT)18 primer and Revert Aid TM first strand cDNA synthesis kit (Fermentas, Hanover, ZMD, USA). Genomic DNA was isolated from young leaves using the genomic DNA purification kit (QIAGEN, Shanghai, China). Primers were designed with the Primer5 software according to the homologous nucleotide sequence in other apple cultivars. High efficiency thermal asymmetric interlaced (high-tail) PCR was also performed to clone promote [53]. The details of the primers are described in Table S2. PLACE and Plant CARE were performed to analysis *cis*-acting elements and binding motif.

### Semi-quantitative PCR analysis

For the semi-quantitative RT-PCR, nucleotide primers were designed according to each gene's conversed region with Primer5, and PCR reactions were performed in final volumes of 25 µL following the thermal profile: 5 min at 95 °C, then followed by 28 cycles of 30 s at 95 °C, 30 s at 56°C and 30 s at 72 °C, a final extension 5 min at 72 °C. Three biological replicates for each sample were provided. *Malus*×*domestica* actin gene (*Mdactin*, Genebank accession number CN938023) as the internal control was used to quantify cDNA abundance. *Arabidopsis TUB2* gene (*TUB2,* Genebank accession number XM_002864767.1) as the internal control was used to quantify cDNA abundance. All of the primers used in this study are listed in Table S3.

### Transformation of *Arabidopsis* and cell callus

The expression analysis was performed. *ZMdPG1* and *anti-ZMdPG1* were recombined into PBI121 vector through *Xba*I and *Bam*HI sites. So they were fused with Gus tag under the control of *CaMV35S* promoter. The *ZMdPG1* promoter fused with *GUS* gene was also recombined into pBI121 vector through *Bam*HI and *Eco*RI sites. Subsequently, they were transformed into *Agrobacterium tumefaciens* strain LBA4404 and introduced into *Arabidopsis* (Columbia O) using the floral dipping method [54]. T1 seeds were selected on half-strength MS medium containing kanamycin (100 mg·L^−1^). After 2 weeks, resistant plants were grown on soil in light incubators under 16/8-h (day/night, 22°C/21°C) photoperiod on matrix. Then T2 seeds were selected as the same method. RT-PCR was also used in T1 line and T2 line of *Arabidopsis* for further verification. Sense *ZMdPG1*, antisense *ZMdPG1* and *ZMdQP* were also transformed into cell callus of ‘Taishanzaoxia’ using dipping method. Resistant materials were selected on MS medium containing kanamycin (100 mg·L^−1^). Primers used for these constructs were shown in Table S4–S5. To investigate expression pattern, GUS histochemical staining assay was performed as described by Sieburth and Meyerowitz [55]. Tissues were fixed, cleared and stained. Then, the stained materials photographed using an Olympus JM dissecting microscope.

### Light microscopy

Tissue was fixed in 4% glutaraldehyde in 100 mM sodium phosphate buffer at pH 7.0, vacuum infiltrated for 30 minutes, and incubated at 4°C overnight. Tissue was briefly flushed in 100 mM sodium phosphate buffer, pH 7.0, and dehydrated in an ethanol series. Then tissue was run through JB4 (A+C)/ethanol mix (1∶1) and immersed in JB4 (A+C) for two days, finally embedded in JB4 (A+C)+(B). Individual tissue in resin blocks were sectioned (2 µm) on a Leica Ultracut R microtome. The sections were dried onto glass microscope slides and stained with a 2% (w/v) aqueous Toluidine Blue solution for 30 s and dried on a hotplate for observation by light microscopy.

### Subcellular localization

Full-length coding sequences of *ZMdERF1,ZMdERF2,ZMdEIL1,ZMdEIL2,ZMdEIL3* and *ZMdPG1* were cloned into the P-58 vector with GFP tag through *Xcm*I sites [56]. All constructs were transformed into *Agrobacterium tumefaciens* strain LBA4404. Primers used for plasmid construction were presented in Table S3. The *Agrobacterium tumefaciens* strains containing different constructs were incubated in infiltration buffer with 10 mM MES, 0.2 mM acetosyringone, and 10 mM MgCl_2_ to an ultimate concentration of OD600 = 0.5. Subsequently, *Agrobacterium tumefaciens* strains were transferred into the same onion epidermis cells. Plants were placed at 24°C for 48 h before detection of GFP fluorescence. The GFP expression in the onion epidermis cells was examined using a Leica confocal microscope (Deerfield, IL, German). Primers used for these constructs were shown in Table S6.

### Bimolecular fluorescence complementation assay (BiFC)

Full-length coding sequences of *ZMdERF1,ZMdERF2,ZMdEIL1,ZMdEIL2* and *ZMdEIL3* were respectively recombined into the binary YFP BiFC vectors [57], so that they were fused with N- or C-terminal fragment of YFP (nYFP or cYFP), and ZMdERF1/ZMdERF2/ZMdEIL1/ZMdEIL2/ZMdEIL3-nYFP and cYFP-ZMdERF1/ZMdERF2/ZMdEIL1/

ZMdEIL2/ZMdEIL3 plasmids were generated. Primers used for plasmid construction are presented in Table S7. All constructs were transferred into *Atumefaciens tumefaciens* strain LBA4404. After incubation, different combinations were co-infiltrated into the same onion epidermis cells. Onion epidermises were cultured at 24°C for 48 h before detection of YFP fluorescence. The YFP signals were examined in the onion epidermis cells using a Leica confocal microscope (Deerfield, IL, German).

### Electrophoretic mobility shift assays (EMSA)

EMSA was performed by the Lightshift Chemiluminescent EMSA kit (Pierce, Rockford, IL, USA). A 1.6-kb fragment of *ZMdQP* was divided into ten linear DAN fragments (100 bp–200 bp). Then they were labeled using an EMSA Probe Biotin Labeling kit (Pierce). The recombinant His-ZMdERF1 protein was purified with His Trap TM FF crude (GE Healthcare, Sweden). The binding reaction was carried out in final volumes of 20 µL containing 1 pmol of labelled probe, 50 ng of purified protein, 25 mm EPES-KOH (pH 7.5), 100 mm KCI, 0.1 mm ethylene diamine tetraacetie acid (EDTA), 17% glycerol, 1 mm DTT and 4 mg of poly (dI–dC). The reactive solution was incubated at room temperature for 30 min. The mixtures were layered on non-denaturing 6% acrylamide gels to electrophorese in 0.5% TBE buffer for 2 h. Then the DNA was transferred to positively charged nylon membranes in the 0.5% TBE buffer for 2 h (Hybond N^+^; Amersham, Little Chalfont, Buckinghamshire, UK), and the signal was detected with the chemiluminescent nucleic acid detection method (Pierce). Primers used for plasmid construction are listed in Table S8.

## Supporting Information

Figure S1 **Homologous assay of ZMdERFs and ERFs in other species.** (A) Amino acid sequence alignment between ZMdERFs and ERFs in other species. Identical amino acids are highlighted in dark gray and similar amino acids in pink and green. Arrows represent conserved YRG and RAYD elements. The accession numbers of these proteins in the GenBank database are as follows: ZMdERF1(KC128856), ZMdERF2(KC128857), RpERF1(AEQ58797.1), LeERF2(NP_001234308.1). (B) Phylogenetic relationship of ZMdERFs and other ERFs protein. The accession numbers of these proteins in the GenBank database are as follows: AaERF1(AEQ93554.1), AaERF2(JN162092.1),RcAP2(XP_002511013.1),EjERF1(AFG26326), PsERF1b(ACM49848.1),GhERF8(AFB35653.1),AdERF12(ADJ67441.1),GhEREB2(AAX68525),GhEREB3(AAX68526), LeERF2(NM_001247379.1), MdERF1(BAF43419.1), MdERF2(BAF43420.1),NtERF2(Q40479.1),RcERF2(F968116.1), RpERF1(AEQ58797.), ZMdERF1(KC128856),ZMdERF2(KC128857).
(TIF)

Figure S2 **Homologous assay of ZMdILs and EILs in other species.** (A) Comparison of the amino acid sequences of ZMdEILs and EILs in other species. Identical amino acids are highlighted in dark gray and similar amino acids in pink and green. Arrows represent BDI, BDII, BDIII, BDIV and BDV domains. AD represent N-terminal acidic region. PR represent proline-rich region. The accession numbers of these proteins in the GenBank database are as follows: ZMdEIL1(KC128858), ZMdEIL2(KC128859), ZMdEIL3 (KC128860), RpERF1(AEQ58797.1), LeERF2 (NP_001234308.1), NtEIL1(AAP03997.1), AdEIL2(ACJ70675.1), LeEIL3 (NP_001234546.1). (B) Phylogenetic relationship of ZMdEILs and other EILs protein. The accession numbers of these proteins in the GenBank database are as follows: AdEIL2(ACJ70675.1),CmEIL2 (BAB64345.1),DcEIL (BAI44821.1),CsEIL1 (ADI40102.1), NtEIL1(AAP03997.1),pEIL2(ABK35086.1),NtEIL5(AAP04001.1),RcEIN3(XP_002530192.1),VvEIN3(XP_002276380.1), EIN3A (XP_002312841.1),EIN3B (XP_002328098.1),EIN3C (XP_002315400.1),EIN3D(XP_002310961.1), LeEIL3(NP_001234721.1),MdEIL1(ADE41153.1),MdEIL2(ADE41154.1),MdEIL3(ADE41155.1),ZMdEIL1(KC128858), ZMdEIL2(KC128859), ZMdEIL3(KC128860).
(TIF)

Figure S3 **Homologous assay of ZMdPG1 and PGs in other species.** (A) Alignment of the ZMdPG1 protein with other PG proteins. Identical amino acids are highlighted in dark gray and similar amino acids in pink and green. GRO represent Gly-rich octapeptide, GS represent potential glycosylation site. The accession numbers of these proteins in the GenBank database are as follows: ZMdPG1(KC128861), Pgdpg-1(P48978.1), PcPG1 (AB066350.1), PcPG2(AB067641.1), PpPG(x77231), AdPG(AAF71160), ADPG1(NP_191310.1). (B) Phylogenetic relationship of ZMdPG1 and other PG protein. The accession numbers of these proteins in the GenBank database are as follows: ZMdPG1(KC128861), pGDPG-1(P48978.1), PcPG1 (AB066350.1), PcPG2(AB067641.1), PpPG(x77231), AdPG (AAF71160), ADPG1(NP_191310.1), ADPG2(NP_850359.1), QPT2(NP_187454.2), LePG2(NP_001234021.1), LePG1 (225933), CpPG(FJ007644), NtPG1(CAA50335).
(TIF)

Figure S4 **Alignment of ZMdQP and rMdPQ. ZMdQP is the promoter of ZMdPG1 from ‘Taishanzaoxia’.** rMdPQ is the promoter of MdPG1 from ‘Royal Gala’. The accession numbers of these proteins in the GenBank database are as follows: ZMdQP (KC128862), rMdPQ (AF031233.1).
(TIF)

Table S1 **Comparison of ZMdPG1, ZMdERFs and ZMdEILs genes with the apple genome.**
(TIF)

Table S2 **Primers used in PCR amplification.**
(TIF)

Table S3 **Primers used for Semi-quantitative PCR.**
(TIF)

Table S4 **Primers used for transformation of Arabidopsis.**
(TIF)

Table S5 **Primers used for GUS Staining.**
(TIF)

Table S6 **Primers used for Subcellular Localization.**
(TIF)

Table S7 **Primers used for BiFC assay.**
(TIF)

Table S8 **Primers used for EMSA assay.**
(TIF)
