# Supplementary figures and images for: Hypersensitive Ethylene Signaling and ZMdPG1 Expression Lead to Fruit Softening and Dehiscence
Source: PLoS One. 2013 Mar 20;8(3):e58745. doi: 10.1371/journal.pone.0058745 (PMC3604122; doi:10.1371/journal.pone.0058745)

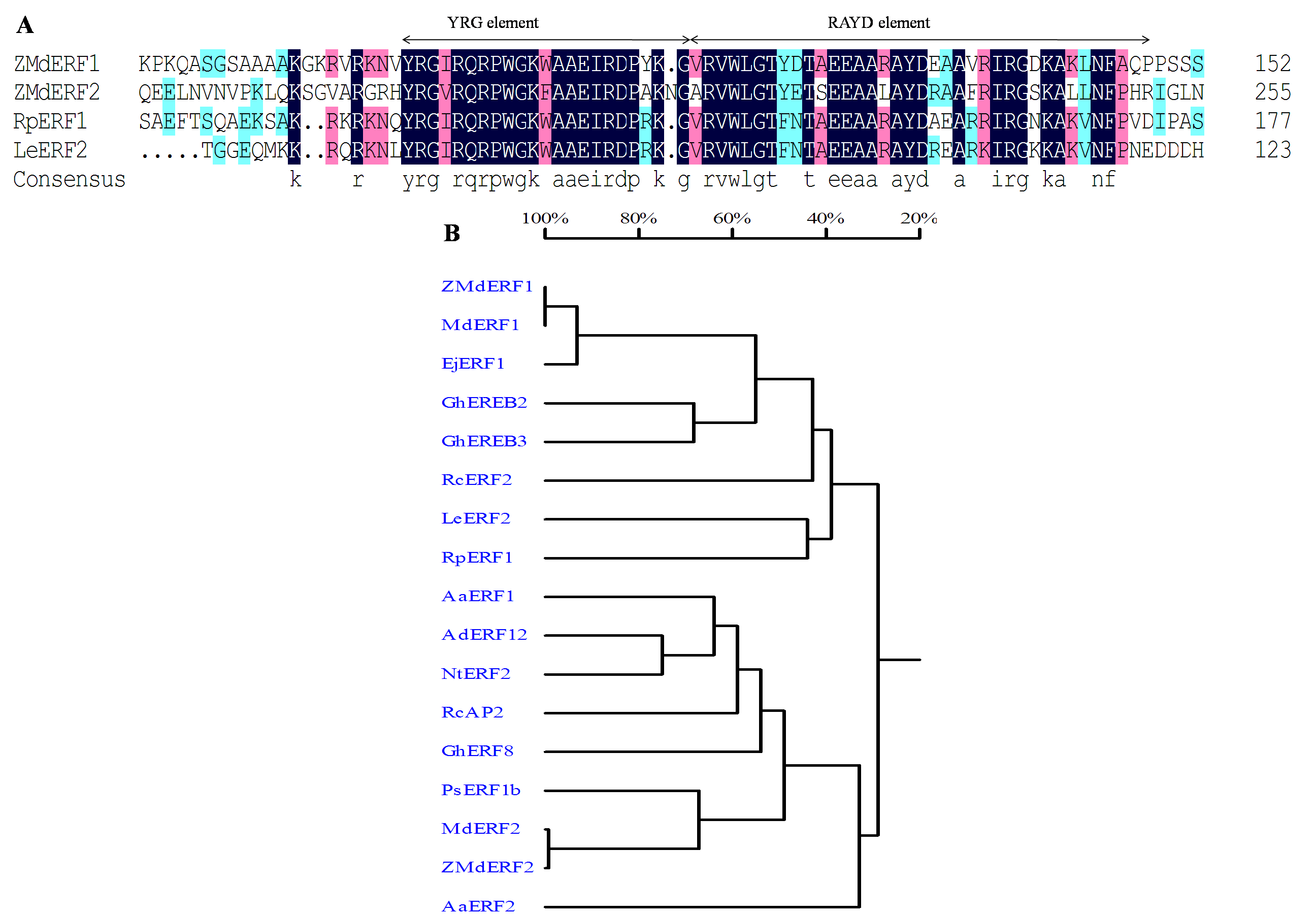

Supplement: Figure S1 — Homologous assay of ZMdERFs and ERFs in other species. (A) Amino acid sequence alignment between ZMdERFs and ERFs in other species. Identical amino acids are highlighted in dark gray and similar amino acids in pink and green. Arrows represent conserved YRG and RAYD elements. The accession numbers of these proteins in the GenBank database are as follows: ZMdERF1(KC128856), ZMdERF2(KC128857), RpERF1(AEQ58797.1), LeERF2(NP_001234308.1). (B) Phylogenetic relationship of ZMdERFs and other ERFs protein. The accession numbers of these proteins in the GenBank database are as follows: AaERF1(AEQ93554.1), AaERF2(JN162092.1),RcAP2(XP_002511013.1),EjERF1(AFG26326), PsERF1b(ACM49848.1),GhERF8(AFB35653.1),AdERF12(ADJ67441.1),GhEREB2(AAX68525),GhEREB3(AAX68526), LeERF2(NM_001247379.1), MdERF1(BAF43419.1), MdERF2(BAF43420.1),NtERF2(Q40479.1),RcERF2(F968116.1), RpERF1(AEQ58797.), ZMdERF1(KC128856),ZMdERF2(KC128857). (TIF) [file pone.0058745.s001.tif]

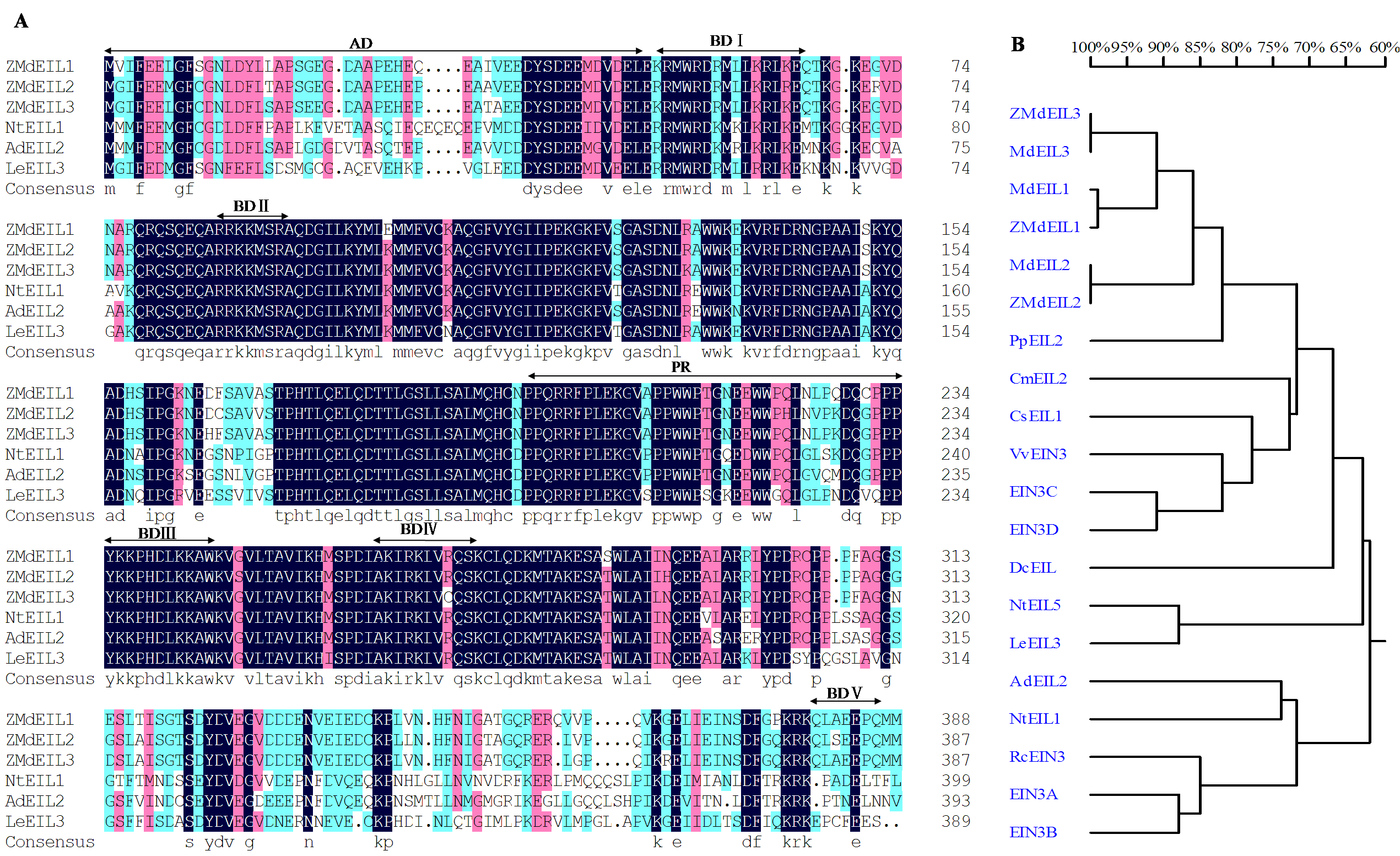

Supplement: Figure S2 — Homologous assay of ZMdILs and EILs in other species. (A) Comparison of the amino acid sequences of ZMdEILs and EILs in other species. Identical amino acids are highlighted in dark gray and similar amino acids in pink and green. Arrows represent BDI, BDII, BDIII, BDIV and BDV domains. AD represent N-terminal acidic region. PR represent proline-rich region. The accession numbers of these proteins in the GenBank database are as follows: ZMdEIL1(KC128858), ZMdEIL2(KC128859), ZMdEIL3 (KC128860), RpERF1(AEQ58797.1), LeERF2 (NP_001234308.1), NtEIL1(AAP03997.1), AdEIL2(ACJ70675.1), LeEIL3 (NP_001234546.1). (B) Phylogenetic relationship of ZMdEILs and other EILs protein. The accession numbers of these proteins in the GenBank database are as follows: AdEIL2(ACJ70675.1),CmEIL2 (BAB64345.1),DcEIL (BAI44821.1),CsEIL1 (ADI40102.1), NtEIL1(AAP03997.1),pEIL2(ABK35086.1),NtEIL5(AAP04001.1),RcEIN3(XP_002530192.1),VvEIN3(XP_002276380.1), EIN3A (XP_002312841.1),EIN3B (XP_002328098.1),EIN3C (XP_002315400.1),EIN3D(XP_002310961.1), LeEIL3(NP_001234721.1),MdEIL1(ADE41153.1),MdEIL2(ADE41154.1),MdEIL3(ADE41155.1),ZMdEIL1(KC128858), ZMdEIL2(KC128859), ZMdEIL3(KC128860). (TIF) [file pone.0058745.s002.tif]

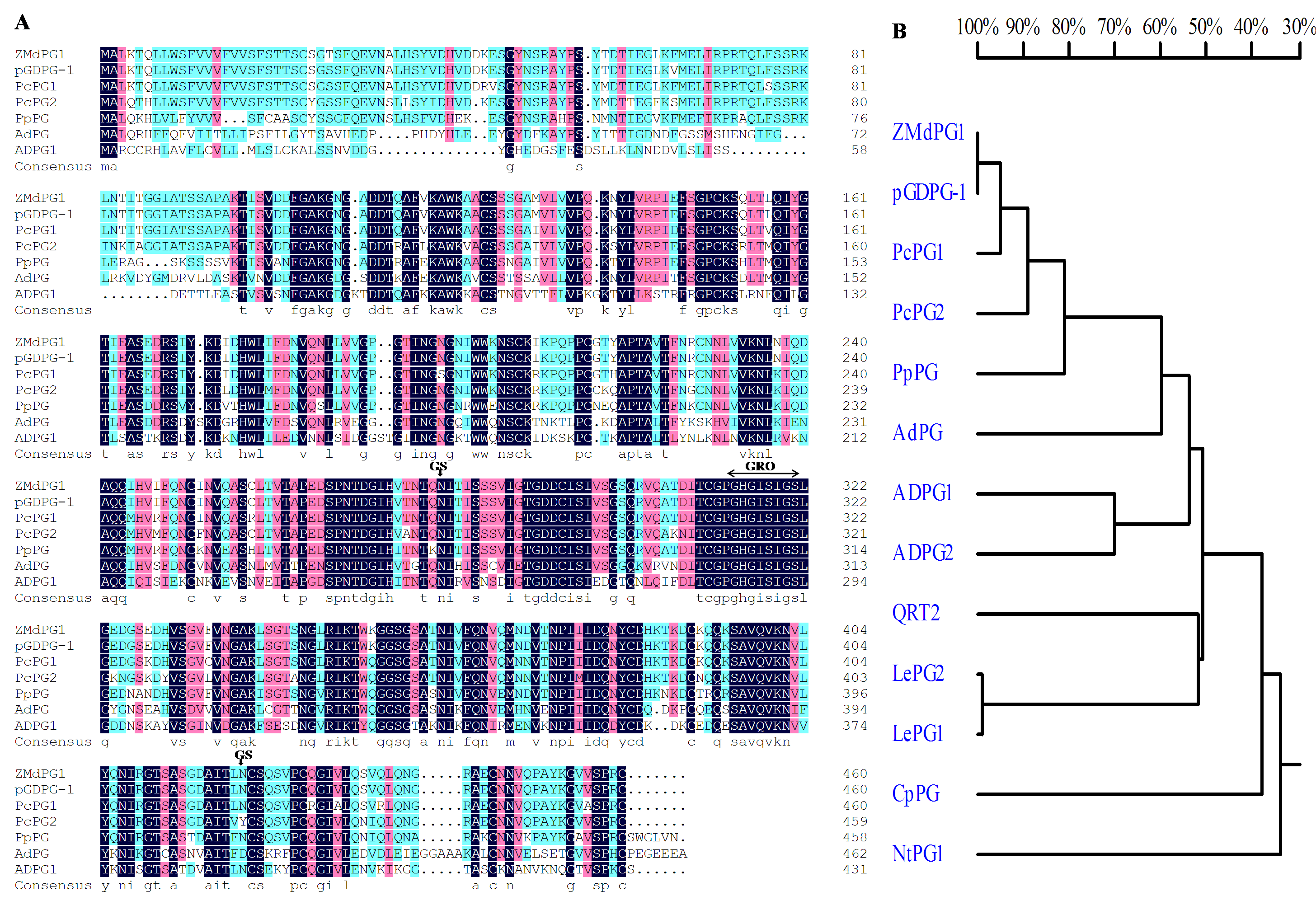

Supplement: Figure S3 — Homologous assay of ZMdPG1 and PGs in other species. (A) Alignment of the ZMdPG1 protein with other PG proteins. Identical amino acids are highlighted in dark gray and similar amino acids in pink and green. GRO represent Gly-rich octapeptide, GS represent potential glycosylation site. The accession numbers of these proteins in the GenBank database are as follows: ZMdPG1(KC128861), Pgdpg-1(P48978.1), PcPG1 (AB066350.1), PcPG2(AB067641.1), PpPG(x77231), AdPG(AAF71160), ADPG1(NP_191310.1). (B) Phylogenetic relationship of ZMdPG1 and other PG protein. The accession numbers of these proteins in the GenBank database are as follows: ZMdPG1(KC128861), pGDPG-1(P48978.1), PcPG1 (AB066350.1), PcPG2(AB067641.1), PpPG(x77231), AdPG (AAF71160), ADPG1(NP_191310.1), ADPG2(NP_850359.1), QPT2(NP_187454.2), LePG2(NP_001234021.1), LePG1 (225933), CpPG(FJ007644), NtPG1(CAA50335). (TIF) [file pone.0058745.s003.tif]

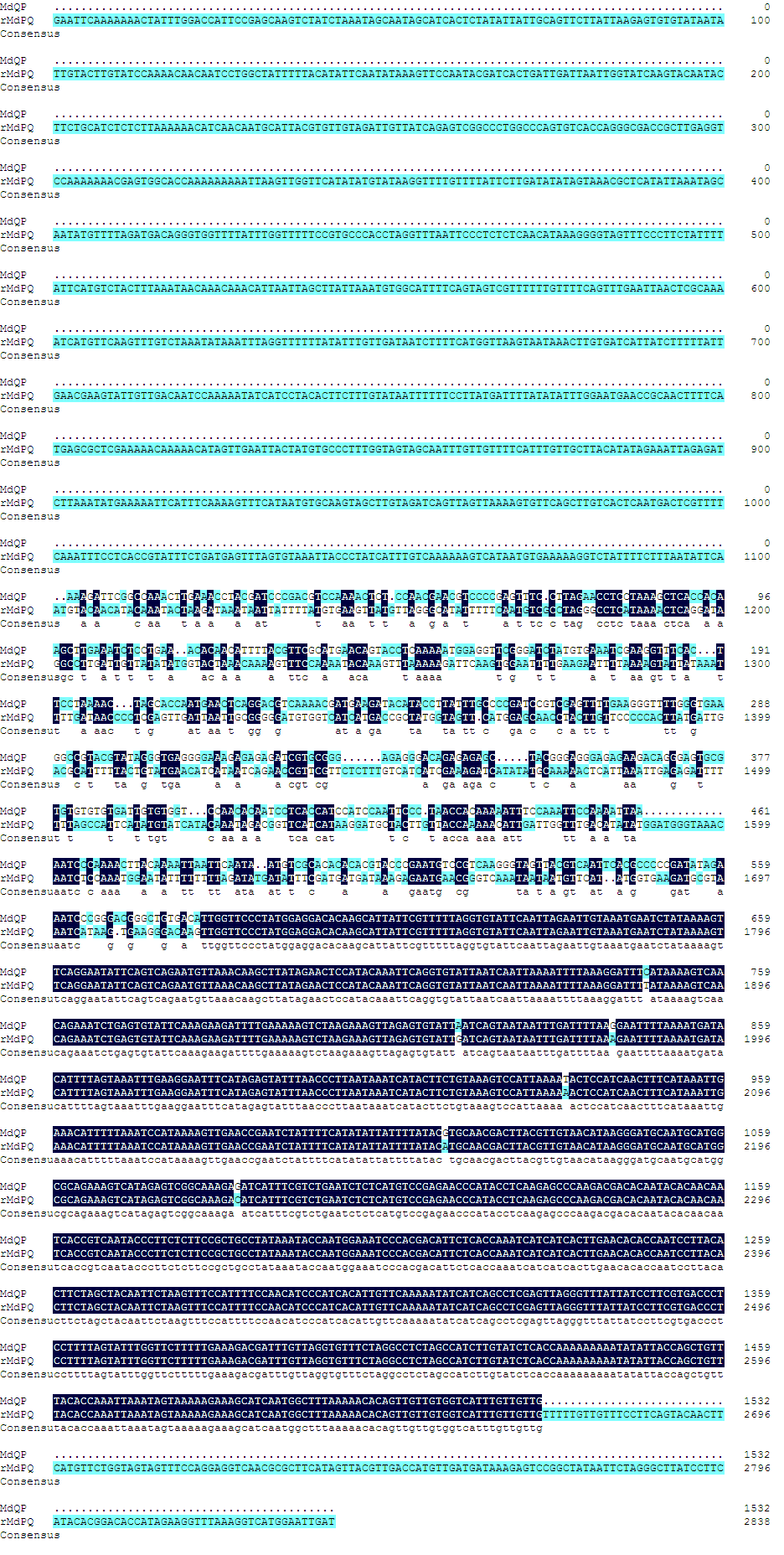

Supplement: Figure S4 — Alignment of ZMdQP and rMdPQ. ZMdQP is the promoter of ZMdPG1 from ‘Taishanzaoxia’. rMdPQ is the promoter of MdPG1 from ‘Royal Gala’. The accession numbers of these proteins in the GenBank database are as follows: ZMdQP (KC128862), rMdPQ (AF031233.1). (TIF) [file pone.0058745.s004.tif]

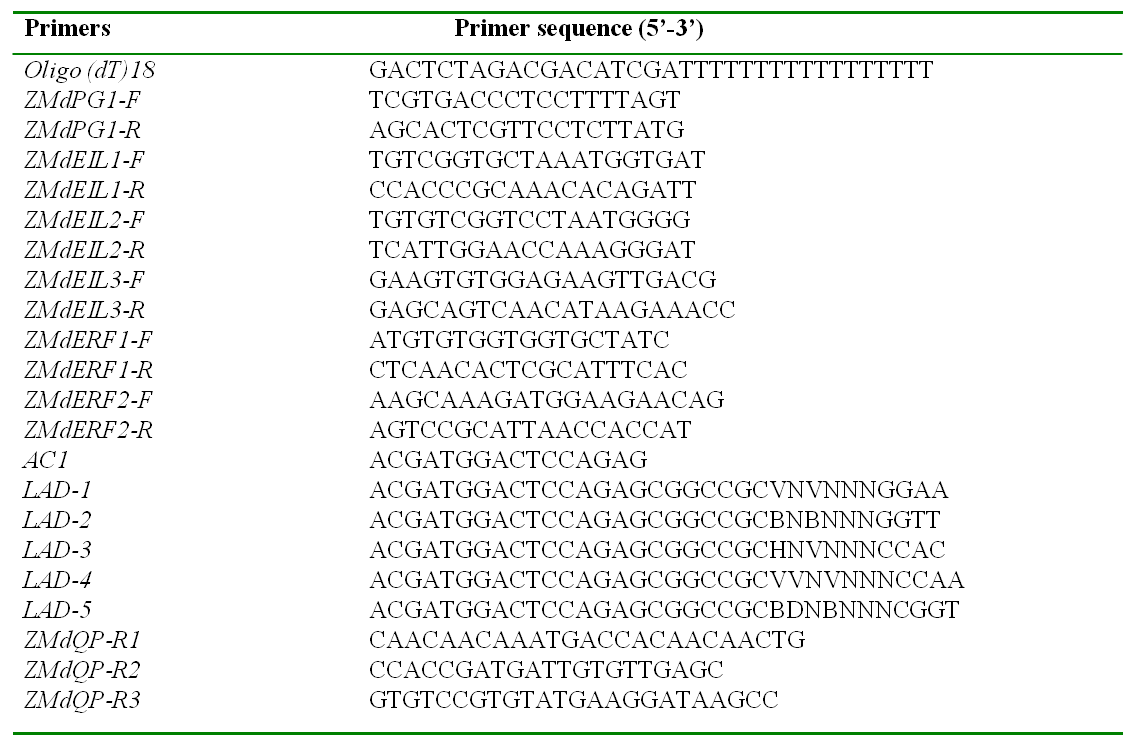

Supplement: Table S2 — Primers used in PCR amplification. (TIF) [file pone.0058745.s006.tif]

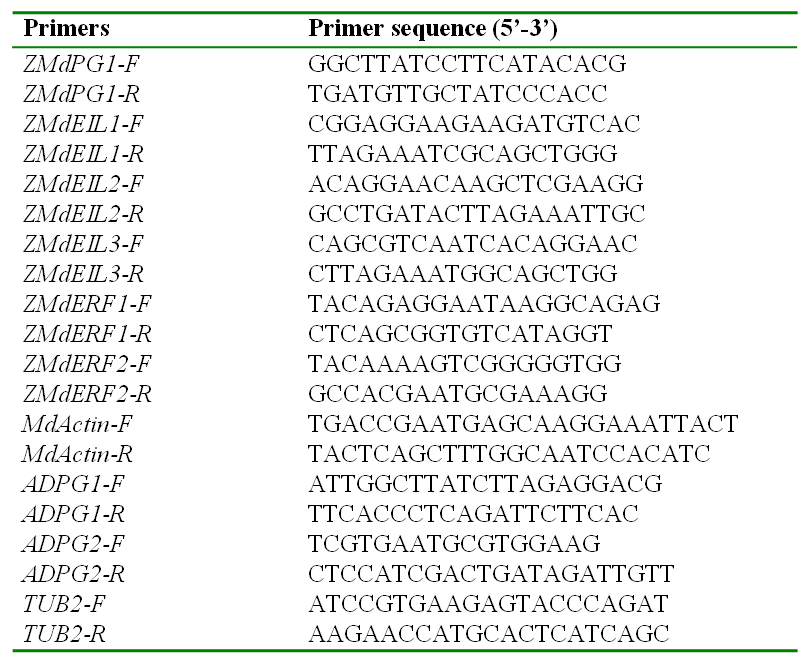

Supplement: Table S3 — Primers used for Semi-quantitative PCR. (TIF) [file pone.0058745.s007.tif]

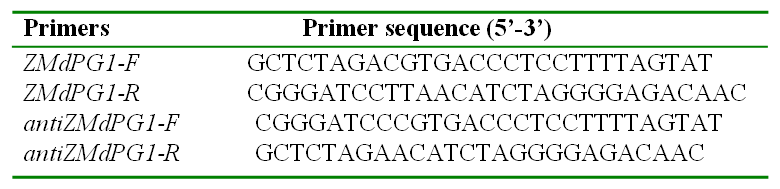

Supplement: Table S4 — Primers used for transformation of Arabidopsis. (TIF) [file pone.0058745.s008.tif]

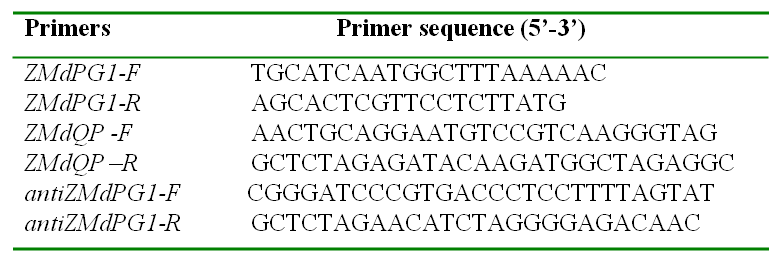

Supplement: Table S5 — Primers used for GUS Staining. (TIF) [file pone.0058745.s009.tif]

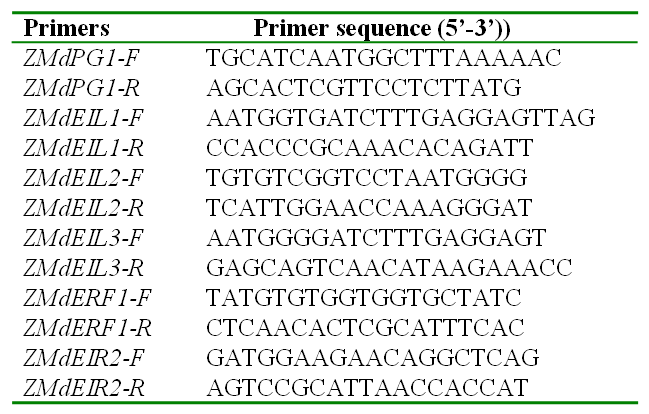

Supplement: Table S6 — Primers used for Subcellular Localization. (TIF) [file pone.0058745.s010.tif]

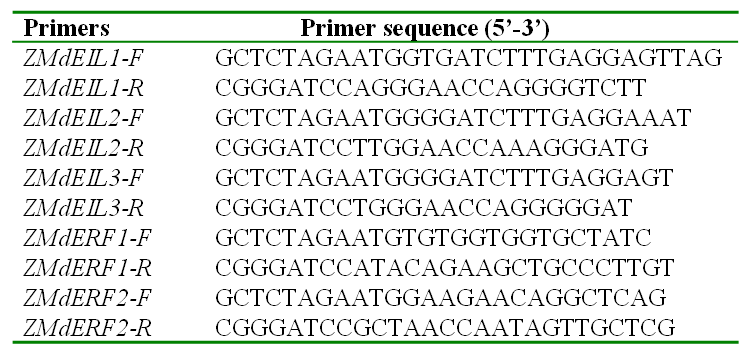

Supplement: Table S7 — Primers used for BiFC assay. (TIF) [file pone.0058745.s011.tif]

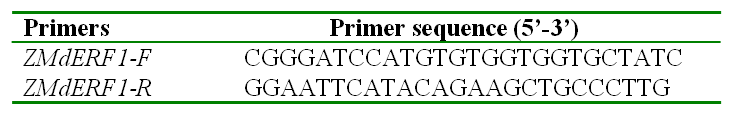

Supplement: Table S8 — Primers used for EMSA assay. (TIF) [file pone.0058745.s012.tif]
